# Supplementary material for: Doxycycline Interferes With Tau Aggregation and Reduces Its Neuronal Toxicity
Source: Front Aging Neurosci. 2021 Mar 22;13:635760. doi: 10.3389/fnagi.2021.635760 (PMC8020845; doi:10.3389/fnagi.2021.635760)
Supplement: Supplementary file 1 [file Data_Sheet_1.PDF]

Supplementary Material

Materials and Methods

Thioflavin T assay

Formation of cross-β structure during tau aggregation was followed by addition of Thioflavin T (ThT) fluorescent probe according to LeVine (LeVine 1999; LeVine 1993) as described in the main text. Doxycycline dose-response assay on tau aggregation was fitted to the equation:

$$IF = \frac{100}{1 + 10^{([Ligand] - \log IC_{50})}}$$

where IF is the normalized fluorescence intensity, [Ligand] is doxycycline concentration, and IC_{50} is the concentration at which aggregation is inhibited at a 50% (Chou 1976; Chou and Talalay 1984).

Doxycycline fluorescence emission spectra

Doxycycline intrinsic fluorescence emission spectrum (Pautke *et al.* 2005) was evaluated in order to detect possible interaction with heparin, which could affect its availability during tau amyloid aggregation.

Emission spectra of 100 µM doxycycline were obtained in the absence and the presence of different heparin concentrations: 0.02 mg/ml, 0.2 mg/ml, 0.3 mg/ml and 0.5 mg/ml.

All emission scans were done using a ISS (Champaign, IL) PC1 spectrofluorometer and a quartz microcuvette under a fixed temperature of 37 °C. Excitation and emission slits were set at 5 nm bandpass. Doxycycline emission spectra in the range of 400–550 nm were acquired with an excitation wavelength of 350 nm.

Supplementary figures and legends

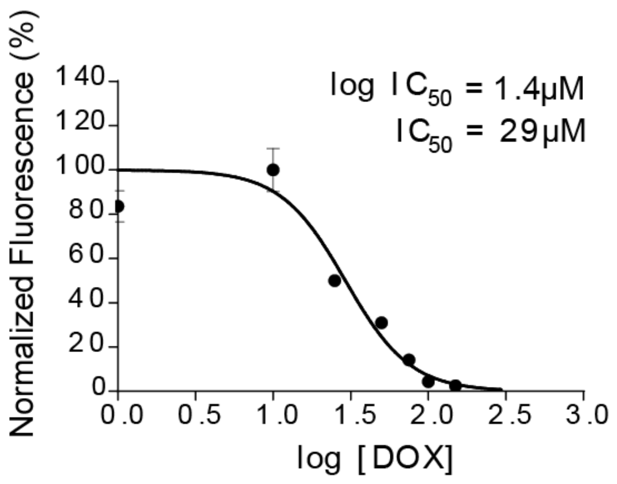

Figure S1. Dose-dependent response of ThT fluorescence of tau:heparin solution (tau 22 µM; 0.2 mg/ml heparin) incubated at 37ºC under orbital agitation for 24 h, in the presence of different concentrations of doxycycline. IC_{50} values are mean values of three independent determinations. The data fitting is described in Methods section.

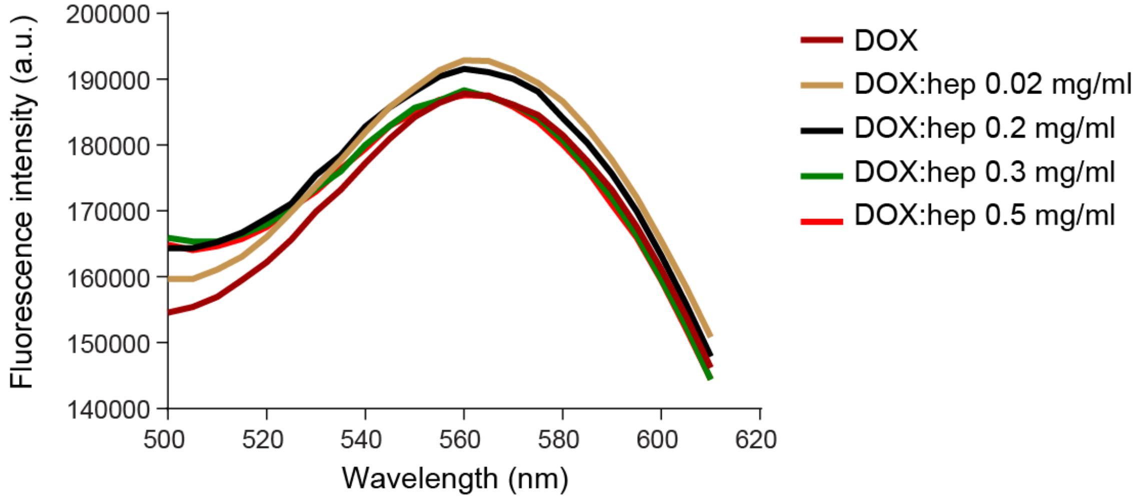

Figure S2. Doxycycline fluorescence emission spectrum is not altered by heparin.

Doxycycline fluorescence emission spectra obtained in the absence and the presence of different heparin doses (0.02 mg/ml, 0.2 mg/ml, 0.3 mg/ml and 0.5 mg/ml) appear in different colors. Very similar emission spectra with the same wavelength peak were obtained, suggesting no direct interaction between doxycycline and heparin.

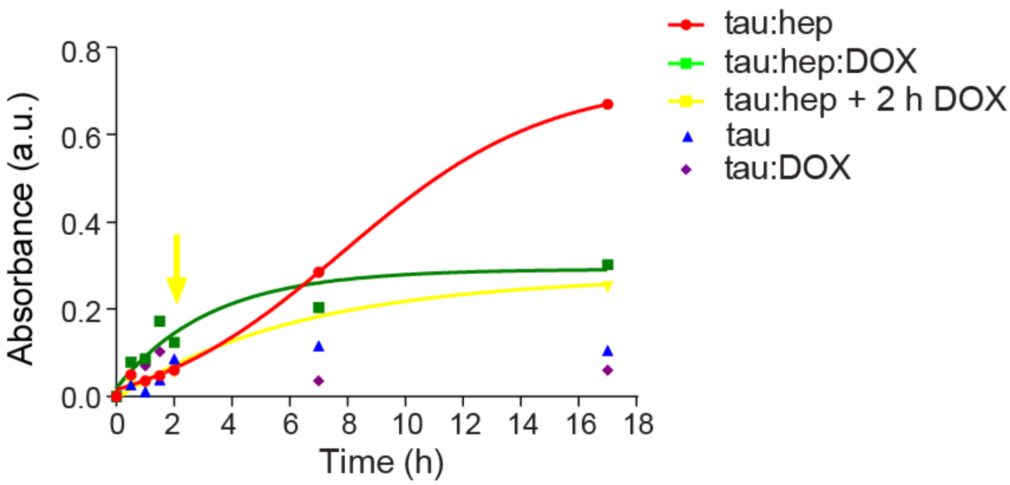

Figure S3. Absorbance of 20 µM Congo Red in a solution containing samples of tau 22 µM; 0.2 mg/ml heparin; and 100 µM of doxycycline added at time 0 h (green line and squares) and at 2 h (yellow line and squares). Samples were incubated at 37°C under orbital agitation, and absorbance recorded over a range of 400–700 nm on a TECAN Infinite M200 microplate reader.

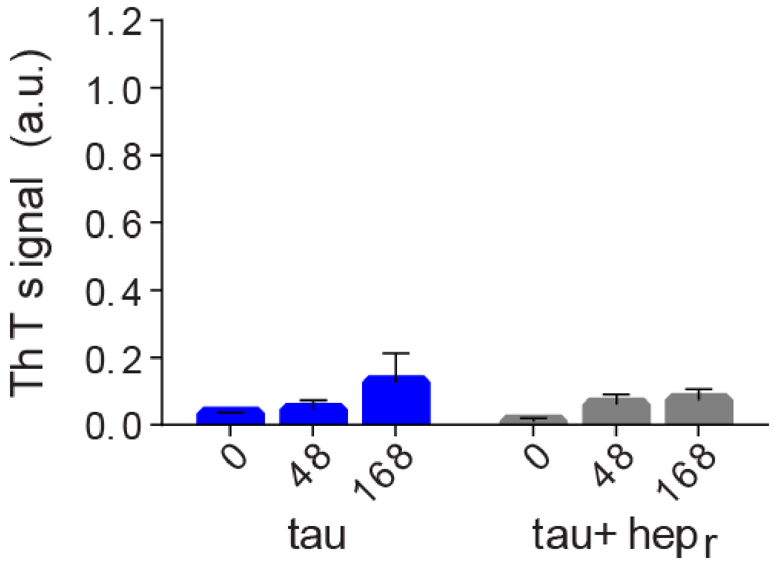

Figure S4. Heparin at the residual concentration has not significant effect on tau aggregation. As an internal control of the seeding assay, heparin at the residual concentration (hep$_r$) (0.08 mg/ml) contained in seed aliquots, was added to monomeric tau and the mix incubated in the same experimental conditions.

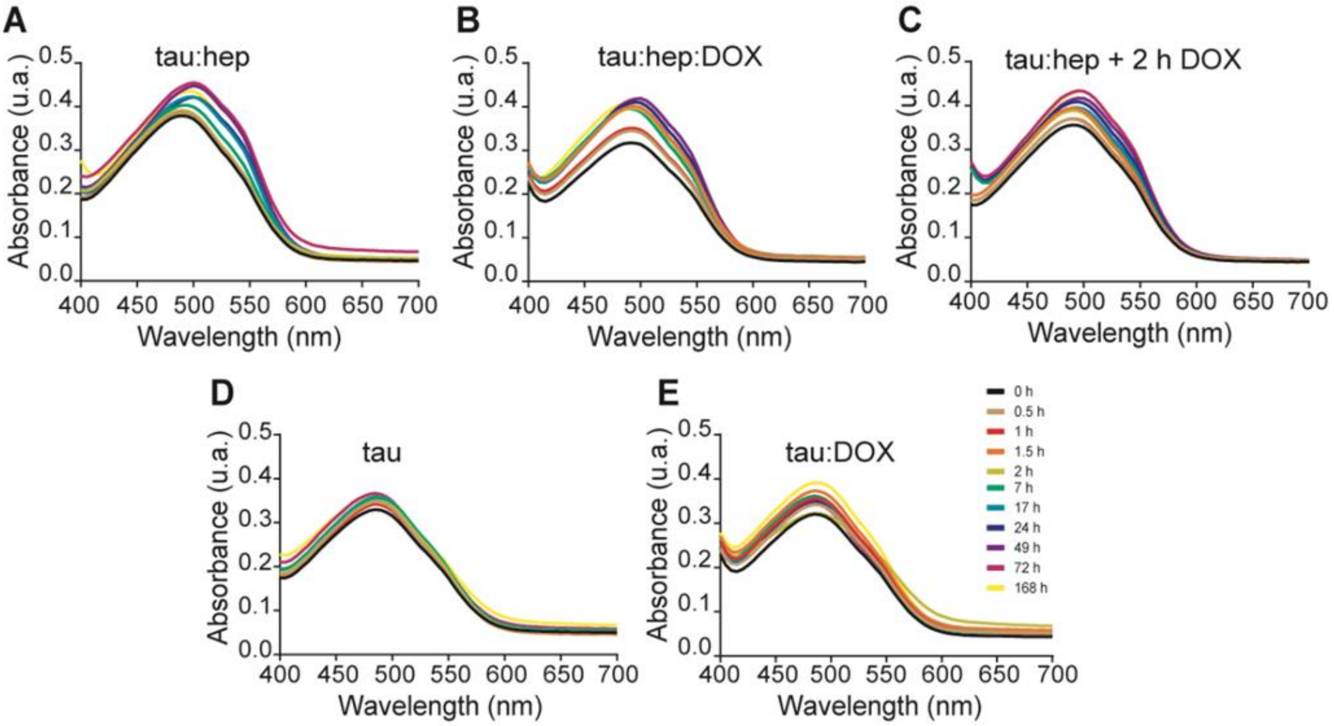

Figure S5. Congo Red Spectra at different times of samples containing tau:hep (A), tau:hep:DOX (B), tau:hep + 2 h DOX (C), tau (D) and tau:DOX (E).

Sample	*Total Protein Content (µg)*		*Percentage of monomer recovered (%)*
	Initial	*Post GnCl*	
**tau**	410 ± 20	380 ± 30	91 ± 7
**tau:hep**	440 ± 10	300 ± 10	69 ± 2
**tau:hep:DOX**	400 ± 10	340 ± 40	85 ± 11

Table S1. Quantification of total protein content. Samples of fresh, monomeric tau (22 µM) were incubated with and without heparin (0.2 mg/ml), in the presence and in the absence of doxycycline (100 µM) for 72 h. Upon incubation, samples were centrifuged and the supernatants were recovered to be treated with guanidine hydrochloride (GnCl). Protein concentration of each sample was measured by DO at 280 nm.

References

Chou T. C. (1976) Derivation and properties of Michaelis-Menten type and Hill type equations for reference ligands. *J. Theor. Biol.* **59**, 253–276.

Chou T. C., Talalay P. (1984) Quantitative analysis of dose-effect relationships: the combined effects of multiple drugs or enzyme inhibitors. *Adv. Enzyme Regul.* **22**, 27–55.

LeVine H. I. (1993) Thioflavine T interaction with synthetic Alzheimer's disease β- amyloid peptides: Detection of amyloid aggregation in solution. *Protein Sci.* **2**, 404–410.

LeVine H. I. (1999) Quantification of beta-Sheet Amyloid Fibril Structures with Thioflavin T. *Methods Enzymol.* **309**, 274–284.

Pautke C., Vogt S., Tischer T., Wexel G., Deppe H., Milz S., Schieker M., Kolk A. (2005) Polychrome labeling of bone with seven different fluorochromes: Enhancing fluorochrome discrimination by spectral image analysis. *Bone* **37**, 441–445.
